
JOURNAL INFORMATION
==============================
NLM Title Abbreviation: Wirtschaftsdienst
Journal ID: Wirtschaftsdienst
NLM Journal ID: 101086612

Wirtschaftsdienst (Hamburg, Germany : 1949)

ISSN: 0043-6275

ARTICLE INFORMATION
==============================
PMCID: PMC8800399
PMID: 35125550
DOI: 10.1007/s10273-022-3096-5
Article ID: 3096
Article version: 1
Subjects: Ökonomische Trends

Coronabedingte Ungleichheit und Armut in Deutschland: Überschätzt oder unterschätzt?

The Rise of Inequality and Poverty in Germany During the Pandemic: False Alarm?

Dauderstädt Michael michael@dauderstaedt.de

Bonn, Deutschland
Electronic publication date: 2022 Jan 29
Print publication date: 2022
Volume: 102
Issue: 1
First page: 64
Last page: 66
Copyright: © Der/die Autor:in 2022
License: Open Access: Dieser Artikel wird unter der Creative Commons Namensnennung 4.0 International Lizenz veröffentlicht (https://creativecommons.org/licenses/by/4.0/deed.de).
Open Access wird durch die ZBW — Leibniz-Informationszentrum Wirtschaft gefördert.
License URL: https://creativecommons.org/licenses/by/4.0/

Keywords: C81, D31, I32
issue-copyright-statement: © The Author(s) 2022

==============================

Anfang November 2021 veröffentlichten sowohl das europäische statistische Amt Eurostat als auch das deutsche Destatis die Ergebnisse der Haushaltsbefragung 2020 zu Einkommen und Lebensbedingungen (EU-SILC: Survey of Income and Living Conditions). Die Ergebnisse für Deutschland waren auf den ersten Blick schockierend: Die Ungleichheit, gemessen mit der Quintilsquote (S80/S20-Quote), also das Verhältnis des Einkommens des reichsten Fünftels der Bevölkerung zu dem des ärmsten Fünftels, stieg von 4,89 auf 6,47. Die Armutsrisikoquote, die den Anteil der Bevölkerung mit einem Einkommen von weniger als 60 % des mittleren Einkommens angibt, kletterte von 17,4 % auf 24,0 %. Das wären über 5 Mio. zusätzlich von Armut(srisiken) betroffene Menschen in Deutschland. Tatsächlich dürfte die Entwicklung weit weniger dramatisch sein; denn Destatis hat 2020 sein Erhebungsverfahren verändert, weswegen die Werte für 2020 mit den Vorjahren nicht vergleichbar sind. Das lässt aber die Fragen offen, wie sich die Einkommensverteilung in Deutschland tatsächlich verändert hat und ob das alte oder das neue Erhebungsverfahren ein realistischeres Bild der deutschen Verhältnisse liefert. Wenden wir uns zuerst der Veränderung in der Pandemie zu.

Vergleicht man Deutschland mit anderen Mitgliedstaaten der EU, so wäre das Land ein kompletter Ausreißer (vgl. Tabelle 1). Auf der Basis von 26 Ländern, für die bei Eurostat Daten vorliegen, sanken (!) die S80/S20-Quote von 4,76 auf 4,68 und die Armutsrisikoquote sank von 21,1 auf 20,58 – im ungewichteten Durchschnitt dieser Länder (ohne Deutschland). Zwar gab es einige Länder mit Zuwächsen (blau markiert in Tabelle 1), aber sie bewegten sich im einstelligen Bereich (außer Malta, wo die Quintilsquote um 12% anstieg, die Armut aber abnahm). In Italien, für das Anfang Dezember 2021 noch keine EU-SILC-Daten vorlagen, dürfte nach einer Schätzung der Banca d'Italia (Carta und De Philippis 2021) die Ungleichheit auch gesunken sein. In Deutschland lagen die Zuwächse bei beiden Indikatoren dagegen bei über 30%!

Angesichts der auch und besonders in Deutschland umfangreichen, wenn auch nicht auf Armutsbekämpfung fokussierten Einkommensstützungsprogramme (Dauderstädt, 2021) ist es wenig plausibel anzunehmen, dass die Entwicklung so stark von den anderen Ländern abwich. Selbst in den USA mit ihrer notorisch hohen Ungleichheit rechnen Studien für 2020 mit einer sinkenden Ungleichheit (JPMorgan, 2021). Wie entwickelten sich Armut und Ungleichheit in Deutschland 2020 nun tatsächlich?

Die Befunde vieler Analysen zu den sozialen Auswirkungen der Corona-Krise in Deutschland geben ein gemischtes Bild: Es gibt einige deutsche Studien, die auf eine Zunahme der Ungleichheit während der Pandemie 2020 hinweisen. Laut Hoevermann und Kohlrausch (2020) gaben Personen mit niedrigerem Einkommen in einer Umfrage (allerdings mit einer begrenzten Stichprobe) höhere Verdienstausfälle an als solche mit höherem Einkommen. Butterwegge (2020) stützt sich auf diese Ergebnisse und befürchtet steigende Einkommensunterschiede und sieht eine höhere Vermögensungleichheit. Bei einer Analyse des deutschen Niedriglohnsektors erwartet Schulten (2020) mehr Armut. Diese Autoren bezweifeln, dass das Kurzarbeitergeld durch den Staat (und die Arbeitgeber:innen) die Einkommen ausreichend stabilisieren kann. Da jedoch noch keine tatsächlichen Daten verfügbar waren, basieren die Ergebnisse auf wenigen Beweisen. Pieper et al. (2021) erwarten einen Anstieg der Armut, der insbesondere arme Rentner:innen betrifft, die nicht von öffentlichen Corona-Hilfsprogrammen profitieren. Untersuchungen des Instituts für Arbeitsmarkt- und Berufsforschung zeigen, dass Geringverdiener:innen stärker von Arbeitslosigkeit betroffen sind als andere Lohngruppen (Buch et al., 2021). Eine Umfrage der Deutschen Bank (2020) findet, dass ärmere Haushalte sich größere finanzielle Sorgen machen. Allerdings gaben nur 24 % an, dass ihr Einkommen gesunken sei, aber bei ärmeren Haushalten lag der Wert bei 27 %, bei den reicheren nur bei 20 %. Auch das WSI (Zucco und Özerdogan, 2021) diagnostiziert größere Probleme bei den einkommensschwachen Haushalten.

Tabelle 1
**Ungleichheit und Armut im EU-Vergleich**

| Land | Quintilsquote (S80/S20) 2019 | 2020 | Veränderung (%) | Armutsrisikoquote 2019 | 2020 | Veränderung (%) |
|---|---|---|---|---|---|---|
| Belgien | 3,61 | 3,65 | 1,11 | 19,5 | 18,9 | -3,08 |
| Bulgarien | 8,10 | 8,01 | -1,11 | 32,8 | 32,1 | -2,13 |
| Dänemark | 4,09 | 4,00 | -2,20 | 16,3 | 15,9 | -2,45 |
| Deutschland | 4,89 | 6,47 | 32,31 | 17,4 | 24,0 | 37,93 |
| Estland | 5,08 | 5,03 | -0,98 | 24,3 | 23,3 | -4,12 |
| Finnland | 3,69 | 3,72 | 0,81 | 15,6 | 16,0 | 2,56 |
| Frankreich | 4,27 | 4,48 | 4,92 | 17,9 | 18,2 | 1,68 |
| Griechenland | 5,11 | 5,15 | 0,78 | 30,0 | 28,9 | -3,67 |
| Irland | 4,03 | 4,13 | 2,48 | 20,6 | 20,9 | 1,46 |
| Kroatien | 4,76 | 4,61 | -3,15 | 23,3 | 23,2 | -0,43 |
| Lettland | 6,54 | 6,27 | -4,13 | 27,3 | 26,0 | -4,76 |
| Litauen | 6,44 | 6,14 | -4,66 | 26,3 | 24,8 | -5,70 |
| Luxemburg | 5,34 | 4,99 | -6,55 | 20,6 | 20,9 | 1,46 |
| Malta | 4,18 | 4,69 | 12,20 | 20,1 | 19,0 | -5,47 |
| Niederlande | 3,94 | 4,04 | 2,54 | 16,5 | 16,3 | -1,21 |
| Österreich | 4,17 | 4,11 | -1,44 | 16,9 | 17,5 | 3,55 |
| Polen | 4,37 | 4,07 | -6,86 | 18,2 | 17,3 | -4,95 |
| Portugal | 5,16 | 4,99 | -3,29 | 21,6 | 19,8 | -8,33 |
| Rumänien | 7,08 | 6,62 | -6,50 | 31,2 | 30,4 | -2,56 |
| Slowenien | 3,39 | 3,32 | -2,06 | 14,4 | 15,0 | 4,17 |
| Slowakei | 3,34 | 3,03 | -9,28 | 16,4 | 14,8 | -9,76 |
| Spanien | 5,94 | 5,77 | -2,86 | 25,3 | 26,4 | 4,35 |
| Schweden | 4,33 | 4,12 | -4,85 | 18,8 | 17,9 | -4,79 |
| Tschechien | 3,34 | 3,34 | 0 | 12,5 | 11,9 | -4,8 |
| Ungarn | 4,23 | 4,30 | 1,65 | 18,9 | 17,8 | -5,82 |
| Zypern | 4,58 | 4,31 | -5,90 | 22,3 | 21,3 | -4,48 |
| Durchschnitt | 4,80 | 4,77 | -0,38 | 20,98 | 20,70 | -0,91 |
| Durchschnitt ohne Deutschland | 4,8 | 4,7 | -1,74 | 21,13 | 20,57 | -2,53 |

Zuwächse sind blau markiert; Quelle: Eurostat; eigene Berechnungen.

Andere Forschungen zeichnen ein positiveres Bild: Eine Studie des Deutschen Instituts für Wirtschaftsforschung (DIW) (Schröder et al., 2020) stellt fest (dort Abbildung 3), dass die Wahrnehmung einer wirtschaftlichen Notlage in unterschiedlichen Einkommensgruppen auf ähnlichem Niveau und deutlich gleichmäßiger verteilt war als in den Vorjahren. Die Anteile der Personen, die steigende, konstante und sinkende Einkommen angeben, sind für alle drei Einkommensterzile sehr ähnlich. In einer Pressemitteilung vom 5. Mai 2021 stellt das DIW fest: „Corona-Pandemie verringert Einkommensungleichheit" (DIW, 2021). Auch Grabka (2021) erwartet, dass in der Pandemie die – langfristig stagnierende – Ungleichheit leicht abnimmt. Eine aktuelle Studie aus dem DIW (Dany-Knedlik und Kriwoluzky, 2021), die langfristig die Auswirkung von Rezessionen auf die Einkommensverteilung untersucht, kommt auf der Basis von Daten aus der World Inequality Database[1] ebenfalls zu dem Ergebnis, dass die Ungleichheit 2020 abgenommen hat.[2] Das stimmt mit dem bekannten Befund überein, dass auch die Lohnquote in Rezessionen steigt, da die Lohnsumme langsamer fällt als die Profite.

Auch weitere Studien erwarten keine Veränderungen der Einkommensverteilung (Bruckmeier et al., 2020). Für Deutschland sehen sie aufgrund der staatlichen Einkommensstützungsprogramme ganz unterschiedliche Auswirkungen der Pandemie je nachdem, ob man die Verteilung der Brutto- oder Nettoeinkommen betrachtet. Mit einer Simulationsstudie schätzen Bruckmeier et al. (dort Abbildung 1), dass die Bruttoeinkommen im Durchschnitt um 3 % und für die verschiedenen Einkommensdezile zwischen 4,3 % (für das 1. Dezil) und 2,8 % (für das 10. Dezil) sinken, was einen leichten Anstieg der Ungleichheit der Markteinkommen impliziert. Beim verfügbaren Einkommen fallen die Ergebnisse jedoch ganz anders aus: Hier beträgt der durchschnittliche Rückgang nur 0,1 %, wobei die Einkommen in den unteren Dezilen steigen und in den höheren Dezilen sinken (dort Abbildung 2). Clark et al. (2020) verwenden Paneldaten einer Erhebung der Universität Luxemburg und kommen zu dem Ergebnis, dass die Ungleichheit des verfügbaren Einkommens in den ersten neun Monaten des Jahres 2020 in Frankreich, Deutschland, Italien, Spanien und Schweden zurückgegangen ist. Ein Vergleich der EU-SILC Daten mit den Angaben in der World Inequality Database für Deutschland zeigt ebenfalls erhebliche Diskrepanzen (vgl. Tabelle 2). Obwohl die Einkommenskonzepte abweichen, bieten die Veränderungsraten eine Vergleichsmöglichkeit.

Zusammenfassend lässt sich sagen: Das Bild für Deutschland ist mehrdeutig und basiert weitgehend auf schwachen Daten. Während die Ungleichheit der Markteinkommen 2020 eventuell zugenommen hat, hat sich die Verteilung der verfügbaren Einkommen wahrscheinlich nicht wesentlich verändert. Das entspräche der Entwicklung in anderen Ländern mit hohem Einkommen (EU, USA). Aber selbst wenn man davon ausgeht, dass die neuen Zahlen von Destatis Armut und Ungleichheit in Deutschland überschätzen, bleibt die Frage, ob die viel niedrigeren Werte der vorherigen Jahre nicht die soziale Spaltung in Deutschland unterschätzt haben. Dazu sollte ein Blick auf die von Destatis vorgenommenen Veränderungen beim Erhebungsverfahren geworfen werden. Während früher die Haushalte meist telefonisch befragt

1 https://wid.world/data/.
2 Diese Autoren weisen auch in einer Fußnote (17) auf die Diskrepanz zu den Destatis-Zahlen hin, ohne näher darauf einzugehen.

Tabelle 2
**EU-SILC und World Inequality Database (WID) im Vergleich für Deutschland**
Veränderung von 2019 auf 2020 in %

| Indikator | EU-SILC | WID |
|---|---|---|
| Einkommen im untersten Quintil | -15,6 | -4,5 |
| Einkommen im obersten Quintil | 11,7 | -6,1 |
| S80/S20-Quote | 32,3 | -1,7 |

Quelle: Eurostat/EU-SILC und WID; eigene Berechnungen.

wurden, konnten sie 2020 die Fragebogen online ausfüllen. Im Gegensatz zu Vorjahren war die Beantwortung nicht mehr freiwillig, sondern Pflicht. Die Befragung wurde in den Mikrozensus integriert, womit sich die Stichprobe in Größe und Zusammensetzung veränderte.

Auf Rückfrage des Autors gab Destatis an, dass sich bei der früheren freiwilligen Erhebung für EU SILC wegen sinkender Teilnahme ein Mittelstandsbias entwickelt habe. Somit würde die neue Erhebung ein realistischeres Bild der sozialen Lage zeichnen. Sie würde besser die Situation von jungen Haushalten und solchen mit Migrationshintergrund abbilden, wodurch sich die Armutsbefunde verstärken. Diese Defizite ergänzen sich mit schon länger bekannten Mängeln, wobei vermutet wird, dass Haushaltsbefragungen wie EU-SILC die Ungleichheit unterschätzen (Bartels und Metzing, 2019). Folgt man diesen Einschätzungen, so wären die eingangs gestellten Fragen wohl wie folgt zu beantworten: Die neuen Zahlen überschätzen die Veränderungen von Armut und Ungleichheit in Deutschland im Zuge der Pandemie. Tatsächlich dürfte der Anstieg, wenn es ihn überhaupt gab, deutlich niedriger als 30 % ausgefallen sein. Aber dies ist kein Anlass zur Entspannung. Denn es sieht so aus, als hätten die früheren Daten die soziale Spaltung in unserer Gesellschaft – in einem allerdings schwer abzuschätzenden Maße – unterschätzt.

Recent data from EU-SILC and Destatis seemed to indicate a dramatic increase in German inequality and poverty in the pandemic year 2020. But Destatis had changed its data collection method. Based on several studies, it is likely that the actual rise has been much weaker while the previous data collection by Destatis might have underestimated the true disparities within German society in the past.

Dr. Michael Dauderstädt ist freiberuflicher Publizist und Berater. Bis 2013 leitete er die Abteilung Wirtschafts- und Sozialpolitik der Friedrich-Ebert-Stiftung.


Literatur

Bartels C Metzing M An integrated approach for a top-corrected income distribution Journal of Economic Inequality 2019 17 125 143 10.1007/s10888-018-9394-x
Bruckmeier, K., A. Peichl, M. Popp, J. Wiemers und T. Wollmershäuser (2020), Covid-19-Krise: Für das Jahr 2020 ist mit keinem Anstieg der Einkommensungleichheit in Deutschland zu rechnen, ifo Schnelldienst digital, 16, https://www.ifo.de/publikationen/2020/aufsatz-zeitschrift/covid-19-krise-fuer-das-jahr-2020-ist-mit-keinem-anstieg-der, (2. November 2021)
Buch T Hamann S Niebuhr A Roth D Sieglen G Arbeitsmarkteffekte der Corona-Krise — Sind Berufsgruppen mit niedrigen Einkommen besonders betroffen? Wirtschaftsdienst 2021 101 1 14 17 10.1007/s10273-021-2818-4 33487768 PMC7813606
Butterwegge C Mehr sozioökonomische Ungleichheit durch Corona? Wie das Virus die Verteilungsverhältnisse beeinflusst Gesellschaft, Wirtschaft, Politik 2020 69 4 493 500 10.3224/gwp.v69i4.08
Carta, F. und M. De Philippis (2021), The impact of the COVID-19 shock on labour income inequality: Evidence from Italy, Occasional Papers, 606, Questioni di Economia e Finanza.
Clark, A. E., C. d’Ambrosio und A. Lepinteur (2020), The Fall of Income Inequality during COVID-19 in Five European Countries ECINE (Society for the Study of Economic Inequality), Working Paper Series, 565, http://www.ecineq.org/milano/WP/ECINEQ2020-565.pdf (2. Dezember 2021).
Dany-Knedlik G. und A. Kriwoluzky (2021), Einkommensungleichheit in Deutschland sinkt in Krisenzeiten temporär, DIW Wochenbericht, 46.
Dauderstädt M Wirtschaftsprogramme gegen die Pandemiekrise — Deutschland im internationalen Vergleich Wirtschaftsdienst 2021 101 5 362 368 10.1007/s10273-021-2917-2 34024949 PMC8127425
Deutsche Bank (2020) Robuste Deutsche? Wie die Bundesbürger die Corona-Krise meistern, https://www.db.com/files/documents/newsroom/Deutsche-Bank-Studie—Wie-die-Bundesbuerger-die-Corona-Krise-meistern.pdf?language_id=3 (15. Dezember 2021).
DIW (2021), Corona-Pandemie verringert Einkommensungleichheit, Pressemitteilung, 5. Mai, https://www.diw.de/de/diw_01.c817355.de/corona-pandemie_verringert_einkommensungleichheit.html (19. November 2021).
Grabka, M. (2021), Income inequality in Germany stagnating over the long term, but decreasing slightly during the coronavirus pandemic, DIW Weekly Report, 17+18, https://www.diw.de/documents/publikationen/73/diw_01.c.817500.de/dwr-21-17-1.pdf (19. November 2021).
Hoevermann, A. und B. Kohlrausch (2020): Soziale Ungleichheit und Einkommenseinbußen in der Corona-Krise — Befunde einer Erwerbstätigenbefragung, WSI-Mitteilungen,73(6).
JPMorgan Chase & Co. (2021), How did the distribution of income growth change alongside the hot pre-pandemic labor market and recent fiscal stimulus?, https://www.jpmorganchase.com/institute/research/household-income-spending/how-did-the-distribution-of-income-growth-change-alongside-the-hot-pre-pandemic-labor-market-and-recent-fiscal-stimulus, (13. November 2021).
Pieper J Schneider U Schröder W Bilanz nach einem Krisenjahr: Corona und die Armut in Deutschland Soziale Sicherheit 2021 1 37 41
Schröder, C., J. Goebel, M. M. Grabka, D. Graeber, M. Kroh, H. Kröger, S. Kühne, S. Liebig, J. Schupp, J. Seebauer und S. Zinn (2020), Vor dem Covid-19-Virus sind nicht alle Erwerbstätigen gleich, DIW aktuell, Nr. 41, https://www.diw.de/documents/publikationen/73/diw_01.c.789499.de/diw_aktuell_41.pdf (2. November 2021).
Schulten, T. (2020), Der Niedriglohnsektor in der Corona-Krise, Aus Politik und Zeitgeschehen, 39–40, https://www.bpb.de/apuz/315575/der-niedriglohnsektor-in-der-corona-krise#footnode9-9 (2. November 2021).
Zucco, A. und A. Özerdogan (2012), Verteilungsbericht 2021. Die Einkommenssituation und Abstiegsängste der Mittelschicht, WSI Report, 69, November, https://www.wsi.de/de/faust-detail.htm?produkt=HBS-008182 (19. November 2021).
